# A 10-Year Follow-Up Study on the Success Rate of Maxillary Sinus Floor Augmentation and Implant Placement in Relation to Strontium Ranelate

**DOI:** 10.3390/dj13120565

**Published:** 2025-12-01

**Authors:** Eliza Dragan, Mihaela Ghinea, Danisia Haba, Gabriel Melian

**Affiliations:** 1 Department of Oral Surgery, Apollonia University, 11 Pacurari Str., 700511 Iasi, Romania; elizadragan@gmail.com; 2 Private Practice, 40 High St., Higham Ferrers NN10 8BL, UK; 3 Department of Surgery, Faculty of Dentistry, Grigore T. Popa University of Medicine and Pharmacy, 16 Universitatii Str., 700115 Iasi, Romania; dhaba@umfiasi.ro (D.H.); gabi.melian@gmail.com (G.M.)

**Keywords:** maxillary sinus floor augmentation, strontium ranelate, bone density, osseointegration, histomorphometry, implant stability, bone remodeling

## Abstract

**Background:** Maxillary sinus floor augmentation (MSFA) is frequently required for implant placement in the atrophic posterior maxilla. However, limited bone quality and volume can compromise long-term success. Strontium ranelate (SrR), a dual-acting bone agent, stimulates osteoblasts while inhibiting osteoclasts, potentially improving bone density and osseointegration in grafted sites. **Objective:** This 10-year preliminary split-mouth study evaluated the long-term effects of SrR on bone density, volume, and implant success following MSFA. **Methods:** Six patients underwent bilateral MSFA using a lateral window approach. One side received systemic SrR (2 g/day for 6 months) after grafting, while the contralateral side served as a control. CBCT and DEXA analyses were performed to assess bone density and volume. Bone biopsies were examined histologically and by microindentation. Data were analyzed using paired t-tests or Wilcoxon signed-rank tests, depending on distribution, with significance at *p* < 0.05. **Results:** After 10 years, SrR-treated sites demonstrated a mean 22.9% increase in bone density versus 12.5% in untreated controls. Although both groups experienced minor reductions in bone volume (SrR: −13.3%; control: −12.8%), SrR samples exhibited greater mineralization, hardness, and lamellar bone maturity. **Conclusions:** SrR improved bone density and mechanical properties but not long-term volume preservation. Given the small sample size (*n* = 6) and absence of implant stability and patient-reported outcomes, these results should be interpreted with caution. Future large-scale clinical trials incorporating survival, ISQ, and quality-of-life data are warranted.

## 1. Introduction

Insufficient bone height in the posterior maxilla, often caused by alveolar resorption and sinus pneumatization, remains a major challenge for successful implant placement. Maxillary sinus floor augmentation (MSFA) is a predictable surgical technique that restores bone volume and enables implant rehabilitation in these cases [1,2]. While autogenous bone remains the “gold standard” because of its osteogenic, osteoconductive, and osteoinductive properties, drawbacks such as donor-site morbidity, unpredictable resorption, extended healing time, and limited availability have prompted the development of alternative grafting materials [3,4].

Strontium (Sr) is a natural trace element that shares chemical similarities with calcium and has been shown to enhance bone formation while inhibiting bone resorption [5]. Strontium ranelate (SrR), a systemic dual-acting bone agent used in osteoporosis therapy, promotes osteoblast differentiation and suppresses osteoclast activity [6,7]. Experimental and clinical research has suggested that SrR may improve bone quality and implant osseointegration when incorporated into grafting protocols [8]. However, limited evidence exists regarding its long-term effects in sinus floor augmentation procedures.

Knowledge gap and study rationale: Although short-term studies have indicated that SrR may enhance bone density and regeneration, few have examined its influence over a decade-long period or compared its performance against conventional sinus augmentation without pharmacological support. Furthermore, little is known about how SrR affects the mechanical quality of regenerated bone over extended periods.

Hypothesis: It was hypothesized that systemic administration of SrR following MSFA would enhance bone density and mechanical strength, leading to improved osseointegration compared with untreated controls.

Objective: The aim of this 10-year preliminary split-mouth study was to evaluate the effects of SrR on bone density, volume, and histological characteristics of grafted maxillary sinuses and to assess its potential as a pharmacological adjunct to traditional augmentation procedures.

## 2. Materials and Methods

### 2.1. Study Design and Ethical Approval

This was a prospective, 10-year preliminary split-mouth clinical study designed to evaluate the effects of systemic strontium ranelate (SrR) on bone regeneration following maxillary sinus floor augmentation (MSFA). The study was conducted at the Department of Oral Surgery, Apollonia University, Iași, Romania. Ethical approval was obtained from the institutional review board (Approval No.61/28 November 2023), and written informed consent was obtained from all participants prior to enrolment, in accordance with the Declaration of Helsinki.

### 2.2. Patient Selection

Six patients (three women and three men; age range: 46–64 years; mean age: 55.3 years) presenting with bilateral partial edentulous in the posterior maxilla were included. Each patient had an alveolar ridge height of 3–5 mm adjacent to the sinus cavity. Exclusion criteria included metabolic bone disease, systemic osteoporosis, smoking, or medication affecting bone turnover.

### 2.3. Study Protocol

Each patient underwent two separate sinus augmentation procedures, spaced six months apart, using a split-mouth design. The first surgery was performed on the right maxillary sinus, followed by a six-month regimen of oral strontium ranelate (Osseor, Servier Pharma, Paris, France; 2 g/day). The contralateral sinus was treated without SrR administration and served as the control.

### 2.4. Surgical Procedure

A lateral window MSFA technique was employed in all cases. A full-thickness mucoperiosteal flap was elevated, and the Schneiderian membrane was carefully reflected. The graft material (Bio-Oss^®^, Geistlich Pharma AG, Wolhusen, Switzerland; 1–2 mm granules) was mixed with autologous peripheral blood.

Antibiotic protocol: A single intravenous dose of 80 mg gentamicin was administered preoperatively as prophylaxis against infection. Gentamicin use was specific to this experimental protocol to ensure graft site sterility and is not part of standard MSFA practice.

Tetracycline administration: Tetracycline (1 g/day for three days before surgery) was prescribed to label newly formed bone, allowing histological identification of mineralization activity under fluorescence microscopy.

After graft placement, the lateral window was closed with a collagen membrane, and the flap was sutured. Postoperative care included amoxicillin/clavulanate (875/125 mg, twice daily for 5 days), chlorhexidine mouth rinses, and analgesics as needed.

### 2.5. Radiographic and Clinical Evaluation

**Figure 1:**
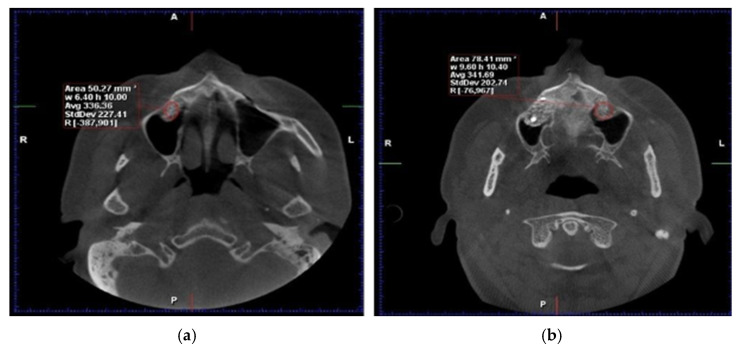
Axial CBCT reconstructions. Evaluation of HU density six months post−intervention: (**a**) with RS administration, (**b**) without adjuvant therapy involving RS (Personal Collection).

**Figure 2:**
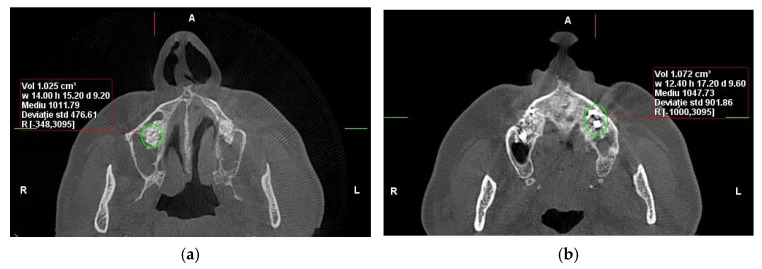
CBCT reconstructions in the axial plane were used to assess bone density (HU) in the area of sinus augmentation at the 10-year follow-up: (**a**) with RS administration, (**b**) without adjuvant therapy involving RS (Personal Collection).

Cone-beam computed tomography (CBCT) and dual-energy X-ray absorptiometry (DEXA) scans were obtained preoperatively, immediately postoperatively, and at 6 months to assess bone volume and density. After six months of healing, bone core biopsies were collected during implant site preparation, and dental implants (Tag Medical Ltd., Kibbutz Ga’aton, Israel) were placed. A follow-up evaluation was performed 10 years after implant placement to assess long-term outcomes, including implant survival and patient-reported satisfaction. The primary analysis in this study was based on imaging and biopsy results collected within the first six months. (Figure 1). However, the 10-year follow-up data focused on long-term implant success and patient-reported outcomes rather than additional CBCT or biopsy examinations (Figure 2).

### 2.6. Histological and Mechanical Analysis

**Figure 3:**
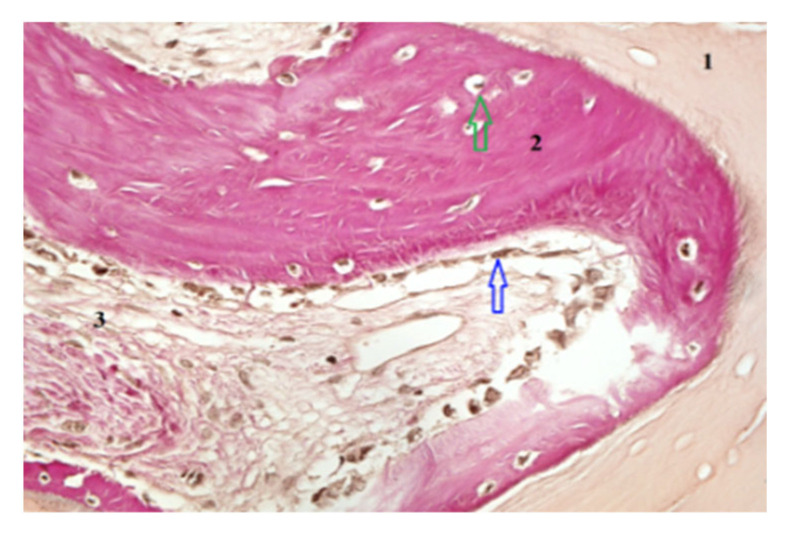
Histological section obtained following RS administration, showing graft particles (1), an osteoblastic reaction (blue arrow) associated with newly formed bone (2) containing osteocytes (green arrow), and regions of stromal tissue (3). Hematoxylin and Eosin staining, magnification 200×.

**Figure 4:**
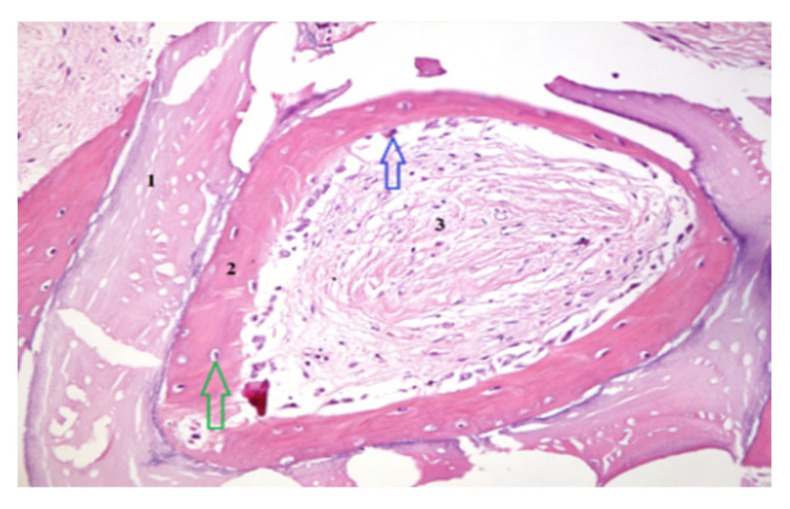
Histological section obtained following RS administration, showing graft particles (1), an osteoblastic reaction (blue arrow) associated with newly formed bone (2) containing osteocytes (green arrow), and areas of stromal tissue (3). VG staining, magnification 400×.

Biopsy specimens were fixed in 10% neutral-buffered formalin, decalcified, embedded in paraffin, and sectioned for Hematoxylin and Eosin (H&E) and Von Gieson (VG) staining. Histomorphometric analysis quantified the percentage of newly formed bone, residual graft particles, and connective tissue using digital microscopy (Zeiss AxioObserver Z1, Viena Austria- Manufactur- Zeiss) and HistoQuest software version 7 (TissueGnostics GmbH, Vienna, Austria). Mechanical properties were assessed by microindentation testing to determine bone hardness and elasticity. For histomorphometric analysis, representative areas of interest—newly formed bone, xenograft particles, and stromal tissue—were carefully outlined at a scale of 400/700 µm. Consistent criteria were applied across the entire patient group to demarcate the boundaries of newly formed bone, xenograft material, and stromal tissue. This ensured reproducibility and accuracy of the measurements for each tissue type (Figure 3 and Figure 4).

### 2.7. Statistical Analysis

All quantitative data were analyzed using IBM SPSS Statistics v27 (IBM Corp., Armonk, NY, USA). Data normality was verified with the Shapiro–Wilk test. Paired *t*-tests were used for normally distributed data (bone density and hardness), while nonparametric Wilcoxon signed-rank tests were applied to non-normally distributed datasets (bone volume and histomorphometric ratios). Statistical significance was set at *p* < 0.05. Given the small sample size (*n* = 6), both tests were used complementarily to ensure analytical robustness. (Table 1)

## 3. Results

### 3.1. Postoperative Outcomes

All patients healed uneventfully following both augmentation procedures. Mild postoperative edema and discomfort were noted but resolved within several days. No cases of infection, sinus membrane perforation, or implant failure occurred during the 10-year observation period. All implants remained functionally stable and clinically successful.

### 3.2. Bone Density

At six months postoperatively, CBCT analysis revealed higher mean bone density in SrR-treated sites compared with untreated controls. Over the 10-year follow-up period, SrR-treated quadrants demonstrated a mean increase of 22.94% in bone density, whereas control quadrants showed a 12.51% increase.

The Hounsfield Unit (HU) values remained consistently higher in SrR sites throughout the follow-up period, confirming a sustained enhancement in mineralization (Table 2).

At 10 years, 66.7% of SrR-treated sites demonstrated a continued increase in density compared with only 20% of controls.

These findings indicate that systemic SrR administration significantly improved bone density and mineral quality over time.

### 3.3. Bone Volume

At six months, bone volume measurements were similar between groups. After 10 years, a gradual decrease was observed in both, consistent with physiological remodeling.

The SrR-treated group exhibited a 13.32% mean reduction in bone volume, whereas the control group showed a 12.82% reduction (Table 3).

Although SrR administration did not prevent volume loss, the rate of reduction was slightly lower in the treated sites, indicating partial preservation of grafted bone architecture.

### 3.4. Histological and Histomorphometric Findings

Histological examination revealed active osteogenesis in both SrR and control sites. However, SrR-treated specimens exhibited more mature lamellar bone, denser trabeculae, and reduced residual graft material.

Quantitative histomorphometry showed that SrR-treated samples contained 46–55% newly formed bone, 15–20% residual graft particles, and 25–30% connective tissue, compared with 35–42% new bone, 25–30% graft material, and 30–35% connective tissue in untreated controls.

The results indicate a higher degree of mineralization and bone maturation in the SrR group. Figure 3 and Figure 4 illustrate these histological differences. Scale bars and tissue labels have been standardized to improve figure clarity.

### 3.5. Mechanical Properties

Microindentation tests demonstrated that SrR-treated bone exhibited superior hardness and elasticity compared with untreated bone. The mean hardness increase corresponded to improved bone microarchitecture and mineral integration. These results align with the higher bone density observed radiographically (Table 4).

### 3.6. Explanation of Missing Data

In Table 5, certain missing values (“?”) correspond to samples in which insufficient bone material was available for microindentation testing due to limited biopsy size. These instances have been clearly indicated in the table footnotes to ensure transparency.

## 4. Discussion

This 10-year split-mouth clinical study evaluated the long-term impact of systemic strontium ranelate (SrR) on bone regeneration following maxillary sinus floor augmentation (MSFA). The findings demonstrate that SrR enhanced bone density and mechanical strength of grafted bone but had a limited effect on long-term volume preservation. Although preliminary due to the small sample size, these results suggest that SrR may serve as a useful pharmacological adjunct for improving bone quality in implantology.

### 4.1. Interpretation of Main Findings

SrR administration produced a mean 22.9% increase in bone density compared with 12.5% in untreated sites, confirming the material’s osteogenic potential. Histological analyses further revealed a higher proportion of lamellar bone and reduced residual graft particles in SrR-treated specimens. The improved microhardness and elasticity observed align with SrR’s known dual mechanism—stimulating osteoblast differentiation while suppressing osteoclast activity [6,7].

Despite these benefits, SrR did not prevent the moderate bone volume reduction observed over the decade. This suggests that while SrR enhances the quality of regenerated bone, it may not significantly influence long-term quantity, which depends on broader biomechanical and remodeling processes.

#### Clinical Implications of Bone Density and Volume Changes

The observed increase in bone density in SrR-treated sites, particularly over the 10-year period, holds significant clinical implications for implantology. Bone density is a critical determinant of implant stability and osseointegration, especially in patients with compromised bone quality. This study’s results demonstrate that SrR can effectively improve bone density over a long period, suggesting its potential as an adjunct treatment to improve implant outcomes, particularly in maxillary sinus augmentations where bone deficiency is prevalent [8,9].

However, despite the positive effects on density, both groups exhibited a decrease in bone volume over time, indicating that bone resorption continues to occur even in the presence of SrR. This highlights the importance of bone volume preservation for long-term implant success. In clinical practice, bone volume loss may compromise implant placement, and SrR alone may not be sufficient to maintain volume over the long term. Adjunctive therapies that enhance bone volume and quality are therefore essential to optimize outcomes in these cases [10].

However, while the SrR-treated group showed improvements in bone density, both groups (with and without SrR) exhibited similar reductions in bone volume over time. Thus, while SrR is beneficial for improving bone quality, its role in preserving bone volume over the long term appears limited. These findings underscore the need for complementary therapies to address bone volume loss, as suggested by Yan et al., who explored the use of bioactive materials alongside SrR for improved bone regeneration [11].

### 4.2. Comparison with Contemporary Technques

Recent innovations in MSFA and ridge reconstruction highlight evolving strategies to minimize grafting and optimize healing.

Menchini-Fabris et al. (2020) proposed distal displacement of the anterior sinus wall as a minimally invasive alternative to the lateral approach, reducing the need for grafting materials [12]. In contrast, the present study focuses on pharmacological enhancement of bone formation rather than surgical modification.

Cosola et al. (2022) demonstrated successful sinus elevation using absorbable collagen alone, underscoring a trend toward graft simplification [13]. The SrR-based approach differs by augmenting osteogenesis biochemically rather than mechanically or by biomaterial reduction.

Crespi et al. (2021) described the split-crest technique for ridge expansion without sinus elevation [14]. When viewed alongside such alternatives, SrR administration represents an adjunctive systemic strategy that could complement rather than replace these minimally invasive surgical methods.

#### Potential Adjunct Therapies to Prevent Bone Volume Loss

Several adjunct therapies have been proposed in recent years to address bone volume loss in sinus augmentation procedures. The combination of SrR with bone grafts, such as xenografts, allografts, or autografts, can potentially provide both structural support and biological stimulation to enhance bone regeneration. Additionally, the use of bioactive materials such as collagen scaffolds and chitosan has been shown to improve cellular attachment and osteogenesis in bone grafts, and combining these materials with SrR may enhance both bone volume and density [15,16]. Growth factors such as Platelet-Rich Plasma (PRP) and Platelet-Rich Fibrin (PRF) have also demonstrated potential in accelerating bone healing and reducing bone resorption during the remodeling phase [17].

Furthermore, bone morphogenetic proteins (BMPs), particularly BMP-2 and BMP-7, have been shown to significantly promote bone formation and regeneration in areas of bone deficiency, suggesting that their combination with SrR could help overcome the limitations of SrR in maintaining bone volume [18,19,20,21,22]. Another promising adjunct therapy is the use of mesenchymal stem cells (MSCs), which are increasingly being used for their osteogenic potential in enhancing bone regeneration and healing [23,24]. Recent studies suggest that combining SrR with additional treatments may help prevent bone volume loss Aroni et al explored the potential of combining SrR with autografts, showing improved bone quality and implant success rates [25].

### 4.3. Additional Biological Considerations

Systemic factors such as vitamin D status can markedly influence bone metabolism, mineralization, and implant osseointegration. Vitamin D deficiency has been associated with delayed bone healing and lower implant stability. Although vitamin D levels were not evaluated in this study, their potential confounding effect warrants consideration in future investigations, as adequate vitamin D supplementation may synergize with SrR to enhance bone regeneration outcomes.

### 4.4. Study Limitations

The main limitation of this study is the small sample size (*n* = 6), which restricts statistical power and generalizability. Furthermore, the absence of quantitative clinical outcomes—such as implant survival rates, implant stability quotient (ISQ) measurements, and patient-reported satisfaction—prevents a comprehensive assessment of long-term functional success. Additionally, the observational design precludes causal inference, and the lack of vitamin D evaluation or standardized dietary control could have influenced the observed variability in bone density.

### 4.5. Clinical Implications and Future Directions

Despite these constraints, the results suggest that systemic SrR may improve the quality of regenerated bone, which could translate to enhanced implant stability in patients with compromised bone. Future research should employ larger, multicenter randomized trials, incorporate detailed clinical outcomes, and explore combined interventions (e.g., SrR with vitamin D supplementation or biologically active graft materials). Longitudinal imaging and mechanical assessments will also be essential to confirm the durability of SrR’s effects beyond the 10-year window presented here.

## 5. Conclusions

Within the limitations of this small preliminary study, the systemic administration of strontium ranelate (SrR) following maxillary sinus floor augmentation enhanced bone density and mechanical strength around dental implants without significantly affecting long-term bone volume preservation. These findings indicate that SrR may serve as a valuable pharmacological adjunct for improving bone quality and osseointegration in the posterior maxilla, particularly in patients with reduced bone density.

However, the restricted sample size (*n* = 6) and the absence of implant stability and patient-reported outcome data limit the generalizability of the results. Future multicenter studies with larger, more diverse populations—incorporating implant survival, stability measurements (ISQ), and quality-of-life indicators—are necessary to validate and expand upon these promising preliminary observations.

## Figures and Tables

**Table 1:** Results of the Wilcoxon Signed-Rank test.

Parameter	The *p*-Value
LL newly bone formed	0.31
LL newly formed stroma	0.84
LR newly formed bone	0.21
LR newly formed stroma	0.68
UL newly formed bone	0.21
UL newly formed stroma	0.21
UR newly formed bone	0.21
UR newly formed stroma	0.15
Percentage of newly formed bone	0.15
Hardness	0.43
Elasticity	0.31

**Table 2:** The evaluation of bone density and volume 6 months postoperatively and at 10-year follow-up.

Sample 6 Month Postoperative	Volume (Quadrant 1 with SR)	Density (Quadrant 1 with SR)	Volume (Quadrant 2 without SR)	Density (Quadrant 2 without SR)
Bi	1.652	579.37	2.023	895.14
Ta	3.507	525.8	1.25	411.18
Cd	1.93	336.36	1.975	341.69
Sg	2.733	597.91	1.682	549.7
TM	2.343	220.81	1.141	661.04
CG	2.84	426.01	1.455	678.28
**Sample** **10-year follow-up**	**Volume (Quadrant 1 with SR)**	**Density (Quadrant 1 with SR)**	**Volume (Quadrant 2 without SR)**	**Density (Quadrant 2 without SR)**
Bi	3.160	820.03	2.469	711.15
Ta	2.934	828.27	0.823	786.14
Cd	1.293	1123.69	1.072	1047.73
Sg	2.946	1011.79	1.254	818.16
TM	1.887	295.49	0.929	744.4
CG	1.969	154.61	1.455	?

Certain missing values (“?”) correspond to samples in which insufficient bone material was available for microindentation testing due to limited biopsy size.

**Table 3:** These results show the percentage changes in volume and density from 6 months to 10 years postoperative for each sample, in both quadrants (with and without Strontium Ranelate). The percentages with a minus sign indicate a decrease in the measured value between the two time points (6 months and 10 years). In other words, a negative percentage means that the value at 10 years is lower than the value at 6 months.

Sample	Volume Change (%) (Quadrant 1 with SR)	Density Change (%) (Quadrant 1 with SR)	Volume Change (%) (Quadrant 2 without SR)	Density Change (%) (Quadrant 2 without SR)
**Bi**	91.46%	41.64%	22.02%	−20.53%
**Ta**	−16.35%	57.54%	−34.16%	90.93%
**Cd**	−32.98%	234.85%	−45.74%	206.69%
**Sg**	7.78%	69.43%	−25.47%	48.83%
**TM**	−19.43%	33.78%	−18.59%	12.61%
**CG**	−30.67%	−63.68%	-	-

**Table 4:** Changes in bone volume and density over 10 years in quadrants with and without ridge splitting (RS).

Quadrant	Metric	Change (10 Years)	Observation
With RS (Quadrant 1)	Volume	−13.32%	Decreased (natural remodeling)
	Density	22.94%	Significant increase
Without RS (Quadrant 2)	Volume	−12.82%	Decreased (similar to RS group)
	Density	12.51%	Moderate increase

**Table 5:** As shown in Table 5, the microindentation tests revealed that the cell density per mm^2^ was 2.59 times higher for small-nucleus cells and 46.03 times higher for large-nucleus cells in the SrR-treated group compared to the non-SrR group. These findings suggest that SrR treatment enhances both the mineralization and mechanical strength of newly formed bone, potentially improving long-term implant stability.

Patient	Quadrant	Volume 6 Months (mm^3^)	Volume 10 Years (mm^3^)	Volume Change (%)	Density 6 Months (g/cm^3^)	Density 10 Years (g/cm^3^)	Density Change (%)
Bi	Quadrant 1 (With RS)	1.652	3.160	91.72%	579.37	820.03	41.50%
Ta	Quadrant 1 (With RS)	3.507	2.934	−16.36%	525.8	828.27	57.63%
Cd	Quadrant 1 (With RS)	1.93	1.293	−32.97%	336.36	1123.69	234.52%
Sg	Quadrant 1 (With RS)	2.733	2.946	7.79%	597.91	1011.79	69.26%
TM	Quadrant 1 (With RS)	2.343	1.887	−19.46%	220.81	295.49	33.74%
CG	Quadrant 1 (With RS)	2.84	1.969	−30.64%	426.01	154.61	−63.70%
Bi	Quadrant 2 (Without RS)	2.023	2.469	22.03%	895.14	711.15	−20.56%
Ta	Quadrant 2 (Without RS)	1.25	0.823	−34.16%	411.18	786.14	91.02%
Cd	Quadrant 2 (Without RS)	1.975	1.072	−45.76%	341.69	1047.73	205.82%
Sg	Quadrant 2 (Without RS)	1.682	1.254	−25.51%	549.7	818.16	49.00%
TM	Quadrant 2 (Without RS)	1.141	0.929	−18.56%	661.04	744.4	12.61%
CG	Quadrant 2 (Without RS)	1.455	1.455	0%	678.28	?	?

## Data Availability

The original contributions presented in this study are included in the article. Further inquiries can be directed to the corresponding author.

## References

[1] Lutz R., Berger-Fink S., Stockmann P., Neukam F.W., Schlegel K.A. (2015). Sinus floor augmentation with autogenous bone vs. a bovine-derived xenograft: A 5-year retrospective study. Clin. Oral Implant. Res..

[2] Spin-Neto R., Stavropoulos A., Coletti F.L., Pereira L.A., Marcantonio E., Wenzel A. (2015). Remodeling of cortical and corticocancellous fresh-frozen allogeneic block bone grafts: A radiographic and histomorphometric comparison to autologous bone grafts. Clin. Oral Implant. Res..

[3] Gentleman E., Fredholm Y.C., Jell G., Lotfibakhshaiesh N., O’Donnell M.D., Hill R.G., Stevens M.M. (2010). Effects of strontium-substituted bioactive glasses on osteoblasts and osteoclasts in vitro. Biomaterials.

[4] Marie P.J., Felsenberg D., Brandi M.L. (2011). How strontium ranelate prevents osteoporosis. Osteoporos. Int..

[5] Meunier P.J., Roux C., Seeman E., Ortolani S., Badurski J.E., Spector T.D., Cannata J., Balogh A., Lemmel E.M., Pors-Nielsen S. (2004). The effects of strontium ranelate on the risk of vertebral fracture in women with postmenopausal osteoporosis. N. Engl. J. Med..

[6] Andersen O.Z., Offermanns V., Sillassen M., Almtoft K.P., Andersen I.H., Sørensen S., Jeppesen C.S., Kraft D.C., Bøttiger J., Rasse M. (2013). Accelerated bone ingrowth by local delivery of strontium from surface-functionalized titanium implants. Biomaterials.

[7] Gazzola D., Beretta M., Bianchi L. (2019). Platelet-rich plasma and platelet-rich fibrin in bone regeneration and implantology. J. Periodontol..

[8] Reis N.T.A., Carvalho J.L.P., Paranhos L.R., Bernardino I.M., Moura C.C.G., Irie M.S., Soares P.B.F. (2022). Use of platelet-rich fibrin for bone repair: A systematic review and meta-analysis of preclinical studies. Braz. Oral Res..

[9] Ressler A. (2022). Chitosan-based biomaterials for bone tissue engineering—A review. Polymers.

[10] Rodríguez J., Escudero N.D., Mandalunis P.M. (2012). Effect of strontium ranelate on bone remodeling. Actaodontol. Latinoam..

[11] Yan M.D., Ou Y.J., Lin Y.J. (2022). Does the incorporation of strontium into calcium phosphate improve bone repair? A meta-analysis. BMC Oral Health.

[12] Menchini-Fabris G.B., Toti P., Crespi G., Covani U., Crespi R. (2020). Distal displacement of the maxillary sinus anterior wall versus conventional sinus lift with lateral access: A 3-year retrospective CT study. Int. J. Environ. Res. Public Health.

[13] Cosola S., Di Dino B., Traini T., Kim Y.S., Park Y.M., Marconcini S., Covani U., Vinci R. (2022). Radiographic and histomorphologic evaluation of the maxillary bone after crestal mini-sinus lift using absorbable collagen. Dent. J..

[14] Crespi R., Toti P., Covani U., Crespi G., Menchini-Fabris G.B. (2021). Maxillary and mandibular split-crest technique with immediate implant placement: A 5-year CBCT study. Int. J. Oral Maxillofac. Implant..

[15] Zhao R., Yang R., Cooper P.R., Khurshid Z., Shavandi A., Ratnayake J. (2021). Bone grafts and bone substitutes in dentistry: A review of current trends and developments. Molecules.

[16] Sakkas A., Wilde F., Heufelder M., Winter K., Schramm A. (2017). Autogenous bone grafts in oral implantology—Is it still a “gold standard”? A systematic review. Int. J. Implant Dent..

[17] Filippi M., Born G., Chaaban M., Scherberich A. (2020). Natural Polymeric Scaffolds in Bone Regeneration. Front. Bioeng. Biotechnol..

[18] Donos N., Mardas N., Boora P. (2023). Bone regeneration in implant dentistry: Which are the factors affecting outcomes?. Periodontology 2000.

[19] Kołodziejska B., Stępień N., Kolmas J. (2021). The influence of strontium on bone tissue metabolism and its application in osteoporosis treatment. Int. J. Mol. Sci..

[20] Tavelli L., Ravidà A., Barootchi S., Chambrone L., Giannobile W.V. (2021). Recombinant human platelet-derived growth factor: A systematic review of clinical findings in oral regenerative procedures. J. Evid. Based Dent. Pract..

[21] Lin X., Wu Y., Huang H., Peng R., Huang F., Hong L., Chen W. (2023). Antibiotic-loaded bone substitutes therapy in the management of moderate to severe diabetic foot infection: A meta-analysis. Wound Repair Regen..

[22] Sukpaita T., Chirachanchai S., Pimkhaokham A., Ampornaramveth R.S. (2021). Chitosan-Based Scaffold for Mineralized Tissues Regeneration. Mar. Drugs.

[23] Gharpinde M.R., Pundkar A., Shrivastava S., Patel H., Chandanwale R. (2024). A comprehensive review of platelet-rich plasma and its emerging role in accelerating bone healing. Cureus.

[24] Giro G., Chambrone L., Goldstein A., Rodrigues J.A., Zenóbio E., Feres M., Figueiredo L.C., Cassoni A., Shibli J.A. (2015). Impact of osteoporosis in dental implants: A systematic review. World J. Orthop..

[25] Aroni M.A., Tampieri A., Berlutti F., Chiesa R., Iafisco M., Prati C., Martini D. (2019). Loading Deproteinised Bovine Bone with Strontium Improves Bone Regeneration in Maxillary Sinus Augmentation: A Preclinical Study. Clin. Oral Implants Res..

